# Repurposing of Sitagliptin- Melittin Optimized Nanoformula against SARS-CoV-2; Antiviral Screening and Molecular Docking Studies

**DOI:** 10.3390/pharmaceutics13030307

**Published:** 2021-02-26

**Authors:** Mohammed W. Al-Rabia, Nabil A. Alhakamy, Osama A. A. Ahmed, Khalid Eljaaly, Ahmed L. Alaofi, Ahmed Mostafa, Hani Z. Asfour, Ahmed A. Aldarmahi, Khaled M. Darwish, Tarek S. Ibrahim, Usama A. Fahmy

**Affiliations:** 1 Department of Medical microbiology and parasitology, Faculty of Medicine, King Abdulaziz University, Jeddah 21589, Saudi Arabia; mwalrabia@kau.edu.sa (M.W.A.-R.); hasfour@kau.edu.sa (H.Z.A.); 2 Department of Pharmaceutics, Faculty of Pharmacy, King Abdulaziz University, Jeddah 21589, Saudi Arabia; nalhakamy@kau.edu.sa (N.A.A.); oaahmed@kau.edu.sa (O.A.A.A.); 3 Center of Excellence for Drug Research and Pharmaceutical Industries, King Abdulaziz University, Jeddah 21589, Saudi Arabia; 4 Mohamed Saeed Tamer Chair for Pharmaceutical Industries, King Abdulaziz University, Jeddah 21589, Saudi Arabia; 5 Department of Pharmacy Practice, Faculty of Pharmacy, King Abdulaziz University, Jeddah 21589, Saudi Arabia; keljaaly@kau.edu.sa; 6 Pharmacy Practice and Science Department, College of Pharmacy, University of Arizona, Tucson, AZ 85704, USA; 7 Department of Pharmaceutics, College of Pharmacy, King Saud University, Riyadh 12372, Saudi Arabia; ahmedofi@ksu.edu.sa; 8 Center of Scientific Excellence for Influenza Viruses, National Research Centre, Giza 12622, Egypt; ahmed_elsayed@daad-alumni.de; 9 College of Sciences and Health Professions, King Saud bin Abdulaziz University for Health Sciences, Jeddah 21582, Saudi Arabia; aldarmahia@ksau-hs.edu.sa; 10 Medicinal Chemistry Department, Faculty of Pharmacy, Suez Canal University, Ismailia 41522, Egypt; khaled_darwish@pharm.suez.edu.eg; 11 Department of Pharmaceutical chemistry, Faculty of Pharmacy, King Abdulaziz University, Jeddah 21589, Saudi Arabia; tmabrahem@kau.edu.sa

**Keywords:** bee venom, nanoparticles, COVID-19, pandemic diseases

## Abstract

The outbreak of the COVID-19 pandemic in China has become an urgent health and economic challenge. The objective of the current work was to evaluate the efficacy of the combined complex of Sitagliptin (SIT) with melittin (MEL) against SARS-CoV-2 virus. SIT-MEL nano-conjugates were optimized by a full three-factor bi-level (2^3^) factorial design. In addition, SIT concentration (mM, X1), MEL concentration (mM, X2), and pH (X3) were selected as the critical factors. Particle size (nm, Y1) and zeta potential (mV, Y2) were assessed as responses. Characterization of the optimized formula for Fourier-transformed infrared (FTIR) was carried out. The optimized formula showed particle size and zeta potential values of 77.42 nm and 27.67 mV, respectively. When compared with SIT and MEL, the combination of SIT-MEL complex has shown anti-viral potential against isolate of SARS-CoV-2 with IC50 values of 8.439 μM with significant improvement (*p* < 0.001). In addition, the complex showed IC50 in vitro 3CL-protease inhibition with IC50 7.216 µM. Molecular docking has revealed that formula components have good predicted pocket accommodation of the SARS-CoV-2 3-CL protease. An optimized formulation of SIT-MEL could guarantee both enhanced delivery to the target cells and the enhanced cellular uptake with promising activities against SARS-CoV-2.

## 1. Introduction

Owing to its epidemic levels, the recent outbreak of COVID-19 in China has emerged as an urgent health and economic challenge [1,2]. There is, therefore, an ongoing race for strategies to treat or prevent COVID-19. The new serious acute respiratory syndrome coronavirus 2 triggers COVID-19 (SARS-CoV-2) [3]. Two other beta-coronaviruses came into prominence over the past 20 years, namely SARS-CoV and Middle East Respiratory Syndrome (MERS)-CoV, albeit without such a pandemic effect [4,5,6]. Likewise, there is growing evidence to suggest that diabetes poses a significant risk factor for the incidence and mortality of COVID-19 in previous influenza infections [7]. A meta-analysis suggested that the most common cardiometabolic comorbidities in COVID-19 hospitalized patients were hypertension, cardiovascular (CV) disease, and diabetes [7]. Arterial hypertension was confirmed by the first large cohort of hospitalized COVID-19 patients in Europe, followed by chronic heart disease and diabetes as the major comorbidities upon hospitalization [8,9], whereas the most common comorbidities were hypertension, obesity and diabetes in the intensive care unit (ICU) [10,11,12]. Moreover, recent studies indicate that even in younger patients, obesity can be associated with increased COVID-19 severity [13,14]. The excessive and prolonged cytokine responses observed in COVID-19 may be directly reduced by sitagliptin (SIT) [15,16]. SIT may enhance glycol-metabolic regulation, which might gain from the clinical progression antagonism of COVID-19 [17]. In patients suffering from type 2 diabetes and COVID-19, impaired glucose regulation has recently been associated with worse outcomes [18,19]. It is notable that the majority of reported deaths occurred in patients aged 70 years and with coexisting pathologies [10,20]. Alterations of age-dependent cellular and humoral immunity may favor increased viral replication and a longer inflammatory response that, in turn, may be responsible for poor mortality outcomes [21,22]. However, some of these changes may be reversed by the SIT inhibitor of DPP4 [12]. Incidentally, solubleDPP-4 plasma levels are upregulated by DPP-4 inhibitors, but this is dissociated from the scope of the inflammatory effect and can be considered as another possible mechanism by which SIT may yield a positive effect [12]. No medication that can be used to treat SARS-CoV-2 infections has currently been developed, which is why the repurposed drug could serve as a favorable alternative for preclinical studies and suitable alternative where development time can be shortened with the potential to effectively combat the disease [17,23,24]. Drugs with high solubility in combination with low permeability are classified into BCS class III. SIT belongs to this class III [25]. The hydrophilic or ionic nature of BCS class III compounds is responsible for their favorable aqueous solubility but potentially jeopardizes their transport across lipophilic biological membranes. Strategies addressing these challenges are needed to improve therapies with hydrophilic compounds. Bee venom is made up of many active ingredients, including enzymes (e.g. phospholipase A2 and hyaluronidase), peptides (e.g. melittin (MEL), apamin, peptide degranulating mast cell and adolapin), amino acids, phospholipids, carbohydrates, biogenic amines, volatile compounds, pheromones, and water (>80%) [26,27,28,29]. Bee venom can cause pain, inflammation and allergic reactions in humans at high concentrations [29]. Additionally, it may activate the immune system’s excessive stress response, which often leads to death [29,30]. On the other hand, low concentrations of this bee product have shown many pharmacological effects in animal models [29]. These include anti-inflammatory, nociceptive, antimicrobial and antitumor effects [31,32]. Interestingly, the replication of the influenza A virus (PR8) and the respiratory syncytial virus was significantly inhibited by the co-incubation of non-cytotoxic bee venom or MEL concentrations, the key component of bee venom [33]. In addition, the distinction between FOXP3-expressing cells in CD4 T cells and mature CD4 thymocytes was significantly enhanced by bee venom [34]. Notably, bee venom has been suggested to induce human regulatory T cell differentiation, which plays a prominent role in the immune response to SARS-CoV infection [34,35]. Further research is needed to explain whether or not the bee venom and its components can be utilized as a SARS-CoV-2 antiviral agent or as a COVID-19 immune system activator [35]. As a matter of fact, chemical strategies such as conjugation with polyethylene glycol (PEGylation), truncation, and peptide synthesis with dextrogenous (d-)amino acids may be able to minimize the toxicity of MEL [36,37]. Such approaches will minimize toxicity of bee venom and allow higher concentrations of this bee product to be used against viral infections. Moreover, by combining these potential anti-COVID drugs with cell-penetrating peptides (CPPs), the potency of these potential anti-COVID drugs can be further improved, which can theoretically improve the cellular uptake of these drugs [38,39,40,41,42,43]. CPPs are short peptides that comprise a strong potential, through energy-dependent and/or independent mechanisms, to cross cellular membranes with peptides themselves show limited toxic effects without the need for chiral recognition by unique receptors [44,45,46,47]. CPPs are efficient vectors for cellular delivery that attract focus on the use of CPPs as molecular transporters [44,48] CPPs that serve as vectors can successfully transport intracellular loads such as small molecules of siRNA nucleic acids and therapeutic agents [44]. The purpose of the work was to examine the efficacy of the combined SIT and MEL complex against the SARS-2 virus. The use of MEL was to augment the uptake and efficacy of SIT. This work within will pave the way for SIT-MEL combination into clinical studies.

## 2. Materials and Methods

### 2.1. Materials

SIT was a gift from Jamjoom pharmaceuticals (Jeddah, Saudi Arabia) and MEL was purchased from Chengdu Youngshe Chemical Co., Ltd. (Chengdu Youngshe Chemical Co., Chengdu, China).

### 2.2. Factorial Design for Development and Optimization of SIT-MEL Nano-Sized Systems

Full three-factor dual level (2^3^) factorial design was implemented to develop SIT-MEL nano-sized systems using Design-Expert^®^ Software Version 12 (Stat-Ease Inc., Minneapolis, MN, USA) (Supplementary file, Figure S1). SIT millimolar concentration (mM, X_1_), MEL millimolar concentration (mM, X_2_), and pH (X_3_) were studied as independent formulation and processing variables, while particle size (PS, nm, Y_1_) and zeta potential (ZP, mV, Y_2_) were recorded as responses, as shown in Table 1. A total of eight formulations were developed in accordance with all possible combinations of the independent variables’ levels (Table 2). The significance of the variables’ effects and their interaction was analyzed using Analysis of Variance (ANOVA) at *p* < 0.05. The coded equation that expresses the chosen factorial model for each response was generated by the software. Further, the desirability function amalgamating the recorded responses to yield a prediction for optimal formulation levels. The goals of optimization were set to minimize the particle size while maximizing the magnitude of the zeta potential (Table 1).

### 2.3. Preparation of SIT-MEL Formulations

SIT-MEL formulations have been prepared according to the aforementioned design. Different SIT and MEL concentrations were put in a 0.01 M phosphate buffer of 20 mL with different pH levels and then vortexed for dissolution for 2 min. To determine the zeta potential and particle size, an aliquot of 1 mL of prepared complexes was further diluted into 10 mL of the same buffer.

### 2.4. Particle Size and Zeta Potential Determination

SIT-MEL size and zeta was investigated using Zetatrac analyzer (Zetatrac; Microtrac Inc., PA, USA). Prepared nanoparticles (NPs) of SIT-MEL formulations were dispersed in water (0.5 mL of each formulation was diluted with 10 mL distilled water) and then measured. The average particle size and zeta potential were calculated from three repeated readings.

### 2.5. Optimization of SIT-MEL NPs

In the statistical analysis of outcomes, two-way ANOVA and multiple-response optimization (Minitab software) were applied. In order to confirm the results, a comparison was made between the zeta potential and particle size of the expected optimum formulation and the prepared formulation.

### 2.6. Fourier-Transform Infrared Spectroscopy of the Optimized SIT-MEL NPs

Fourier-transformed infrared (FTIR) was used for the interaction investigation between SIT and MEL spectra, calculated using an FTIR spectrophotometer between 4000–400 cm^−1^ (Nicolet IZ 10, Thermo Fisher Scientific, Waltham, MA, USA).

### 2.7. IC_50_ Calculation Using Crystal Violet Assay

The assay was performed according to the procedure previously described with minor modifications [49]. Vero E6 cells were seeded into 96-well plates in 100 μL of DMEM Complete Medium containing DMEM high glucose medium with 2 mM L-glutamine, 1 mM sodium pyruvate, and 10% fetal bovine serum (FBS), 100 units/mL penicillin and 100 µg/mL streptomycin. After 24 h (90–100% confluence monolayer of Vero E6), each compound was diluted using infection DMEM in a separate U shape 96 well plate (with a range of concentration from 10 µg/mL to 1 ng/mL) into varying concentrations. An aliquot of 100 µL of each dilution was transferred into new U shape 96 well plate and supplemented with 100 TCID50 in 100 µL infection media. In parallel, the wells dedicated for CC_50_ calculation were supplemented with 100 µL infection media without virus [49].

### 2.8. In Vtro Mpro 3CL-protease Inhibition Test

A fluorescent substrate harboring the cleavage site (indicated by the arrow, ↓) of SARSCoV-2 Mpro (Dabcyl-KTSAVLQ↓SGFRKM-E (Edans); BPS Bioscience, Inc., US), 3C-like protease (SARS-CoV-2 3CL Protease), GenBank Accession No. YP_009725301, a.a. 1-306 (full length), expressed in an E. coli expression system, MW 77.5 kDa., and buffer composed of 20 mM Tris, 100 mM NaCl, 1 mM EDTA, 1 mM DTT, pH 7.3 was used for the inhibition assay, and GC376 a 3CL protease inhibitor, MW 507.5 Da was used as control [50,51]. In the fluorescence resonance energy transfer (FRET)-based cleavage assay, the fluorescence signal of the Edans generated due to the cleavage of the substrate by the 3CL Protease was monitored at an emission wavelength of 460 nm with excitation at 360 nm, using a Flx800 fluorescence spectrophotometer (BioTek). Initially, 30 µL of diluted SARS-CoV-2 3CL Protease at the final concentration of 15 ng was pipetted into a 96-well plate containing pre-pipetted 10 µL of test formula. The mixture was incubated for 30 min at 20 °C with slow shaking. Afterwards, the reaction was initiated by adding 50 μL reaction buffer that contain 10 µL dissolved substrate, at a concentration of 40 μM, incubated for 4 h at room temperature with slow shaking. The plates were sealed. Fluorescence intensity was measured in a microtiter plate-reading fluorimeter capable of excitation at a wavelength 360 nm and detection of emission at a wavelength 460 nm [51].

### 2.9. Molecular Docking Study

Using the Molecular Operating Environment (MOE; 2019) software program was deemed beneficial for conducting the presented molecular docking studies. This comprehensive platform permits construction of the investigated ligands, preparation of the biological target, carrying out of molecular docking simulation, and analysis of the predicted ligand-target interactions [52]. Moreover, the MOE software has multi-disciplinary applications including small Protein-Protein Docking protocol [53,54]. Initially, both SIT and MEL protein were built in MOE software and then energy-minimized through the adopted Merck Molecular Force Field with 2000 steps conjugated gradient approach at 1 × 10^−3^ Kcal/Å gradient. MEL was constructed as helix protein secondary structure geometry using the MOE Protein Builder Module relying on its primary structure (amino acid sequence) being deposited at PubChem database (CID: 16133648). On the other hand, SIT was constructed via MOE Builder tool relying on the deposited isomeric SMILES (PubChem CID: 4369359). The X-ray crystallized structure of SARS-CoV-2 main protease (3-chymotrypsine-like protein hydrolase; 3CLpro) was downloaded from the free on-line RCSB-Protein Data Bank database (PDB code: 6lu7) at 2.16 Å atomic resolution. Using the MOE module for protein preparation, the target protein was protonated and auto-corrected for types of atoms, bond connectivity, and partial charges [55,56]. Defining the active binding site was proceeded through the MOE Alpha-Site Finder tool, subsequently refined for including the reported residues being important for 3CLpro substrate binding and guidance within the active pocket. The defined pocket was of 109 in size where this value indicates the number alpha spheres comprising this site. The alpha sphere is the geometric feature of the protein’s Voronoi diagram used to map out concave interaction [57,58]. An alpha sphere is a sphere that contacts 4 atoms on its boundary while containing no internal atoms. Using (Maestro^®^ version 12.0.012, release 2019-2), a surface area of 728.811 Å^2^ was assigned for the defined binding site being estimated at 5 Å from the crystallized ligand. The propensity-for-ligand-binding score for the contact residues within the pocket was the highest (3.05), which is generally estimated through summing up the RA of all site residues [59]. Typically, RA is derived from a database of high-quality protein-ligand complex structures and has a constant value for each type of amino acid. Important pocket residues included within the assigned site; like the catalytic dyad His41 and Cys145 as well as Met49, Tyr54, Phe140, Leu141, Asn142, Gly143, Ser144, His163, His164, Met165, Glu166, Leu167, Asp187, Arg188, Gln189, Thr190, and Gln192 lining the 3CLpro substrate binding (Figure S1). Docking of the prepared minimized ligands was proceeded by adopting the triangle matcher approach where the docking scores of the generated obtained binding modes were obtained and ranked via the London-dG scoring function [60,61,62]. Docking poses were then refined through energy minimization within the pocket before being rescored via the GBVI-WSA/dG force field scoring system, which depends on the Coulombic’s and solvation electrostatic, van der Waals score, exposure-weighed surface area, as well as solvation electrostatic [63]. Selection of relevant pose was based on obtaining the highest final docking score. For further validation of the obtained pose, the authors refined the top-ranked poses, having close final docking scores, based on the RMSD value of the docked ligand relative to the crystallized N3. The more preferential docking pose was with lower RMSD value. A cut-off value below 2.0 Å was assigned for SIT.The PyMol V.2.0.6 software was used for graphical visualization of the predicted poses and analysis of the predicted ligand-binding interactions.

### 2.10. Statistical Analysis

The IBM SPSS^®^ statistical software (Ver. 25, 2017, SPSS Inc., Chicago, IL, USA) software was chosen to conduct the comparative tests one-way variance analysis (ANOVA) was used in consonance with Tukey’s post-hockey test. Each set of experiments reported as a mean ± standard deviation (SD) was performed on at least four occasions. A *p* value < 0.05 has been considered statistically significant.

## 3. Results

### 3.1. Statistical Analysis of the Factorial Design

Identifying the formulation and process factors that could contribute to the drug delivery system attributes is pivotal in the field of developing pharmaceutical formulations. Factorial design proved an important value in this concern as it can concurrently analyze the impact of the studied factors on the measured responses. ANOVA was used to examine the significance of the studied variables. For both responses, the predicted R2 values are in good coincidence with the adjusted R2 values. Adequate precision was greater than 4 (Table 3), affirming that the model is suitable for navigating the experimental design space. For detailed design data see Table S1, design Build Information(Table S2), design factors (Table S3), design responses (Table S4) (data not shown).

### 3.2. Effect of Variables on Particle Size (Y_1_)

Effect of the particle size of the nano-sized delivery systems on their biological performance was proven to be significant in this study. Accordingly, the particle size measurement is a crucial step for developing such systems. The particle size of the prepared SIT-MEL nano-sized systems ranged from 121.31 ± 2.11 to 432.11± 5.12 nm (Table 2). Factorial analysis revealed the significance of factorial model with main effects process order (Model *F*-value = 87.70; *p* = 0.0004). There is only 0.04% likelihood that an *F*-value could be attributed to noise. The coded equation (Equation (1)) elucidating the main effects was generated.

*Particle size (nm)* = 272.14 + 43.85 × (SIT concentration) + 99.79 × (MEL concentration) − 5.85 (pH) (1)

**Figure 1:**
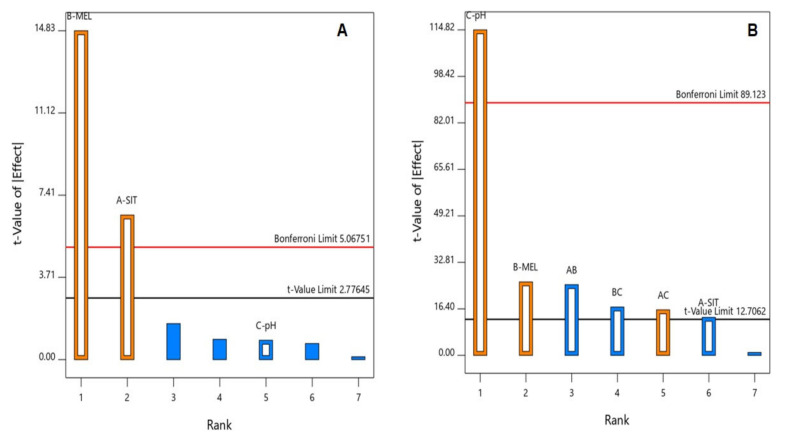
Standardized Pareto chart for the (**A**) particle size and (**B**) zeta potential of SIT-MEL nano-sized systems.

**Figure 2:**
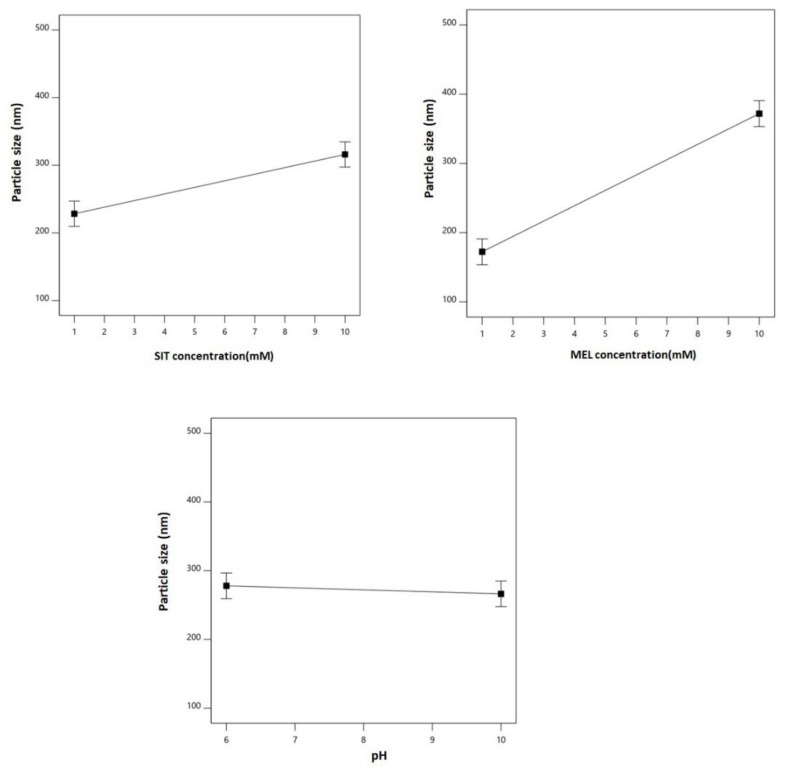
Main effects of SIT concentration (X_1_), MEL concentration (X_2_), and pH (X_3_) on particle size of SIT-MEL nano-sized systems.

Analysis of Variance (ANOVA), using sum of squares Type III-partial showed that both SIT (X_1_) and MEL (X_2_) concentrations demonstrate a positive significant effect on the particle size (*p* = 0.0029 and 0.0001, respectively). This positive effect is supported by the positive sign of the coefficients of both terms X_1_ and X_2_, which are graphically illustrated in a Pareto chart in Figure 1A. Figure 2 graphically illustrates the individual effects of the assessed variables on the particle size. As seen from the figures, the size increases with increase in both SIT and MEL concentrations. For size analysis detailed ANOVA (Table S5).

### 3.3. Effect of Variables on Zeta Potential (Y_2_)

Zeta potential is a reflection for the charge stabilization of nanoparticulate systems. All the prepared SIT-MEL nano-sized systems exhibited positive zeta potential ranging from 6.27 ± 0.33 to 32.25 ± 1.15 (Table 2). According to the factorial analysis, the factorial model with two-factor interaction (2FI) process order was significant at the set level (Model *F*-value = 2533.85; *p* = 0.0152). There is only 1.52% likelihood that an *F*-value could be ascribed to noise. The coded equation (Equation (2)) illustrating the magnitude of the main effects and interactions of the independent variables was generated.

Zeta potential (mV) = 19.94 − 1.12 × (SIT concentration) + 2.17 × (MEL concentration) +9.62 × (pH) − 2.09 × (SIT concentration) × (MEL concentration) + 1.34 × (SIT concentration) × (pH) − 1.43 × (MEL concentration) × (pH)(2)

Analysis of Variance (ANOVA), using sum of squares Type III-partial revealed a significant effect of all the investigated variables; namely, SIT concentration (X1, *p*-value = 0.0475), MEL concentration (X2, *p*-value = 0.0246), and pH (X3, *p*-value = 0.0055) on the zeta potential as illustrated in the Pareto chart (Figure 1B.) Notably, the pH exhibits the highest impact on the zeta potential as supported by its highest coefficient in the coded equation and the lowest *p* value. In addition, all the interaction terms representing the binary interactions between the studied factors were found to be significant at 95% confidence level.

**Figure 3:**
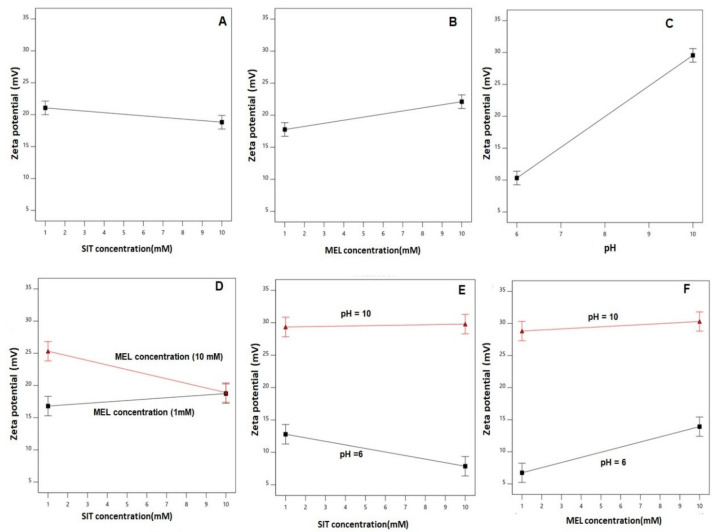
Main effects (**A**–**C**) and interactions (**D**–**F**) of SIT concentration (X_1_), MEL concentration (X_2_), and pH (X_3_) on zeta potential of SIT-MEL nano-sized systems.

The main effects of the explored variables and the two-factor interactions between them on the zeta potential are graphically represented in Figure 3. As evident, the zeta potential values decreased with increased drug concentration, while it goes up with increasing MEL concentration and pH. The graphs of the main effects confirm the marked significance of pH on the zeta potential in comparison to the rest of the variables [64,65,66]. For size analysis detailed ANOVA (Table S6).

### 3.4. Selection of the Optimized SIT-MEL Nano-Sized Systems

The optimal SIT-MEL nano-sized systems was chosen based on the goals set for the responses during the optimization process before computing the desirability function. The formulation developed using SIT concentration of 1.00 mM and MEL concentration of 1.00 mM at pH equal to 10 was found to meet the predetermined criteria being minimum size an maximum zeta potential with a desirability of 0.881. Therefore, this formulation was selected for further biological studies. The chosen formulation was again prepared and assessed for particle size and zeta potential before being subjected to biological studies. The measured particle size and zeta potential were 123.12 nm and 26.99 mV, respectively. These results were in good agreement with the predicted values (122.65 nm and 26.50 mV, respectively), with residual error percentage of less than 1% for both responses.

### 3.5. Fourier-Transform Infrared Spectroscopy Investigation of the Optimized SIT-MEL NPs

**Figure 4:**
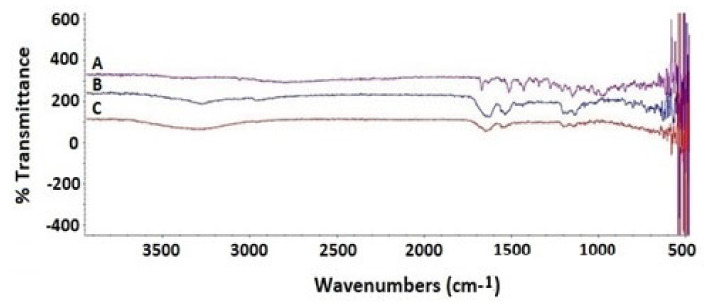
FTIR spectra of SIT (**A**), MEL (**B**) and combination of SIT-MEL (**C**).

SIT base form showed a characteristic band regions that can be assigned in the following manner: 3049 cm^−1^ aromatic C-H stretching, 1650–1690 cm^−1^ is associated with the amidic C=O bond stretching, 1630 cm^−1^ refers to The Imine C=N bond, 1570 cm^−1^ is related to N-H Bending vibration (N–H), 1465 cm^−1^ is related to C–H bending of methylene group, and The vibrations at 1000–1400 cm^−1^ is related to fluoride (C–F) (Figure 4).

MEL showed a very broad band at 3300–3400 cm^−1^ of NH2 stretching of free and amidic amino groups and guanidine groups. MEL spectra were given a broad band range of 1600–1700 cm^−1^ and fitted in the amide group region of peptide backbone and 1500–1600 cm^−1^ that were related to N-H Bending vibration of NH_2_ (Figure 4). The C– O stretch vibrations from the C-terminal amino acid were detected between 1100–1250 cm^−1^. SIT Mel get an increasingly broad peaks of amino group stretching region at 3200–3700 cm^−1^. Moreover, SIT-MEL revealed a sharp decrease in the intensity of peak bands at 1550–1700 cm^−1^ and 1100–1400 cm^−1^ of both SIT and Mel, thus demonstrating the ionic interaction between the negatively and positively charged essential function groups of SIT and Mel, respectively.

### 3.6. Determination of the Antiviral Activity

**Figure 5:**
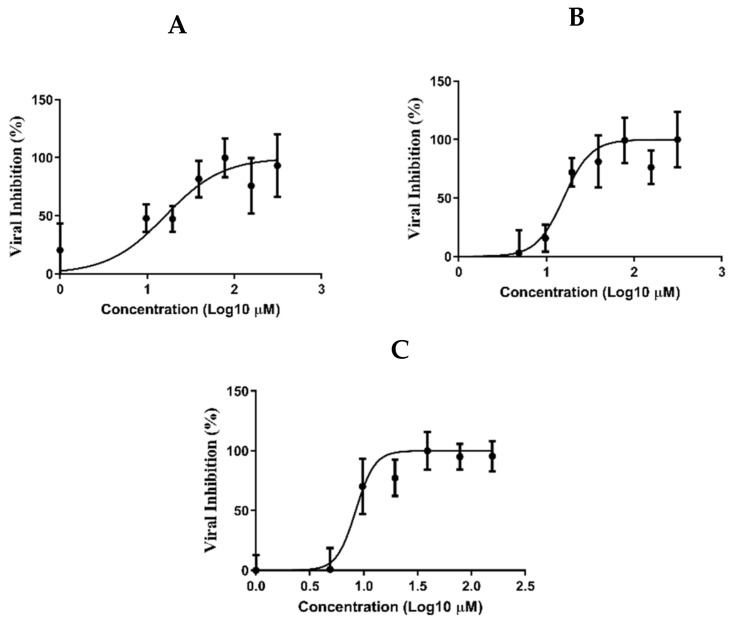
IC_50_ of SIT (**A**), MEL (**B**), and combination of SIT-MEL (**C**) against SARS-CoV-2.

The cytotoxicity of SIT, MEL and combination of SIT-MEL in Vero E6 cells were measured by crystal violet assay as previously described [67]. The result showed that the CC50 values of SIT, MEL and combination of SIT-MEL were non cytotoxic (Supplementary Materials S1 and S2, Figures S2 and S3). The antiviral activities of SIT, MEL and combination of SIT-MEL were determined on the basis of dose–response using crystal violet staining [49]. SIT and MEL showed IC50 values of 16.14 and 15.73 µM, respectively (Figure 5A,B). In comparison to SIT and MEL, SIT-MEL combination (Figure 5C) exhibited a significantly (*p* < 0.001) improved effect (8.439 µM).

### 3.7. In Vitro Mpro 3CL-Protease Inhibition

**Figure 6:**
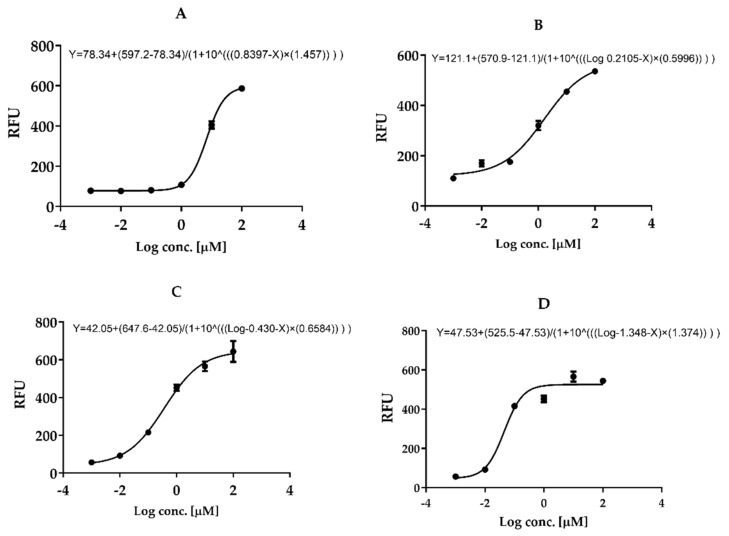
Inhibition of 3CL Protease enzyme activity by SIT (**A**), MEL (**B**), SIT-MEL (**C**) and GC376 inhibitor control (**D**).

Results revealed that in vitro Mpro 3CL-protease inhibition of the complex SIT-MEL (IC50 = 0.3715 µM ± 0.001) (Figure 6C) was significantly enhanced, *p* < 0.001, compared with the individual components SIT (IC50 = 6.914 µM ± 0.034, Figure 6A) and MEL (IC50 = 1.624 µM ± 0.014) (Figure 6B). GC376 (inhibitor control IC50 = 0.04488 µM ± 0.001, Figure 6D). The Hillslopes were 1.457, 0.5996, 0.6584 and 1.374, respectively.

### 3.8. Molecular Docking Study

**Figure 7:**
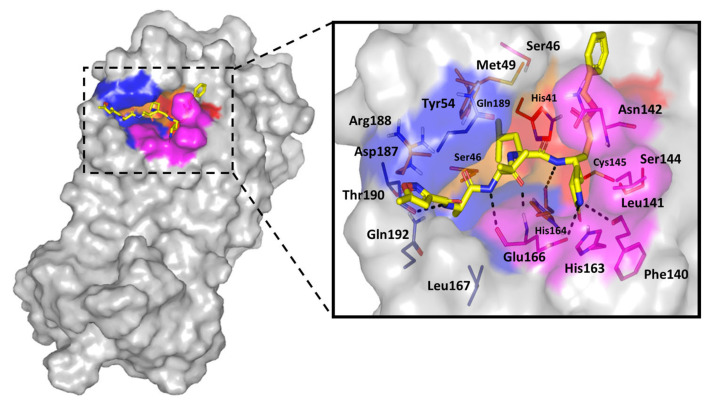
Structure of crystallized SARS-CoV-2 3CL^pro^ target complex (PDB code: 6lu7). Protein target (gray surface) is bound to the crystallized potent irreversible inhibitor (N3; yellow sticks), within the canonical substrate binding site. This shows the four important sub-pockets (S1’, S1, S2, and S3, as red, magenta, orange, and blue color, respectively). Zoomed stereoview of N3 (yellow sticks represent the ligand–protein hydrogen bonding as black dashed-lines. Residues (lines) located within 5 Å radius of bound ligands are colored in accordance with sub-pocket being labeled with sequence numbers.

Based on the furnished compound’s in-vitro inhibition activity on SARS-CoV-2 3CLpro, an in-silico study was established to investigate the differential binding modes of ligands toward 3CLpro active site and identify key residues involved within ligand-target complex interactions [68,69]. Throughout the computational study, docking of the ligands of interest was performed on the SARS-CoV-2 3CLpro (PDB code: 6lu7). The protein is bounded to the irreversible Michael-acceptor peptidomimetic inhibitor, N3, covalently fitting at the substrate active site resembling 3CLpro natural substrate for preventing the enzyme catalytic activity (Figure 7). The crystal complex consists of four sub-pockets (S1’-S4) corresponding to the four peptide partitions (P1’–P4) of natural substrate [70]. Several pocket residues are currently recognized for their significant role in binding of different ligands toward the 3CLpro active pocket [71]. Binding to the S1’ sub-pocket, particularly to His41 and/or Cys145 as the 3CLpro catalytic dyad, is depicted as vital for strong ligand-target binding and 3CLpro hydrolytic activity inhibition. It is also noteworthy that valuable hydrophobic interactions with Met165 and Gln189 side chains, at S3 sub-pocket, as well as His41, Met49, and Asp187 (S2 sub-pocket) can serve as non-polar grip anchoring several ligands toward the binding pocket. Concerning hydrophilic interactions, both the nitrogen and carbonyl of Glu166 main chain (S1 sub-pocket) play a key role in ligand-protein binding. Several authors further reported different residues, including Thr24, Thr25, Pro168, His172, Phe185, and Ala191 to be relevant for selected ligand anchoring [51,72,73].

**Figure 8:**
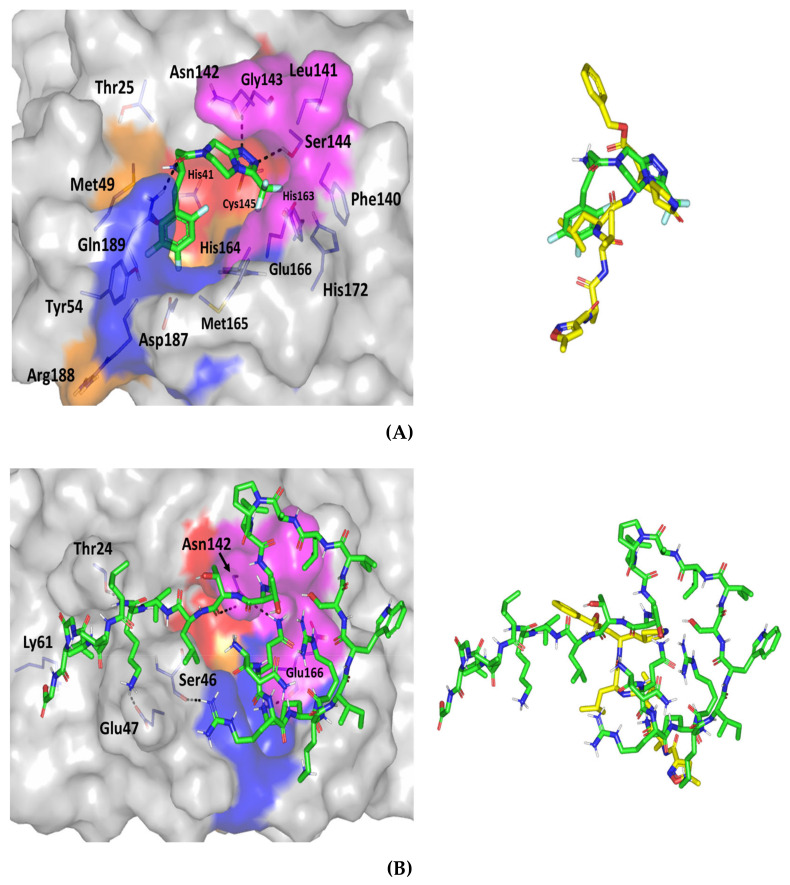
Docking poses sitagliptin (**A**); melittin formulation proteinomimetic ligand (**B**), within the SARS-CoV-2 3CL^pro^ active site (PDB code: 6lu7). Left panels depict the suggested ligand-target complexes showing docked ligands as green sticks, while 3CL^pro^ target (gray surface) with different colored sub-pockets (S1’ = red; S1 = magenta; S2 = orange; S3 = blue). Overlaid conformations of docked ligands (green sticks) and crystallized N3 (yellow sticks) are depicted at rights panels. The ligand–protein hydrogen interactions are represented as black dashed-lines. Residues (lines) located within 5 Å radius of bound ligands are colored in accordance with sub-pocket being labeled with sequence numbers.

With regard to the docking results of the investigated hypoglycemic agent, significant accommodation of the target binding site was depicted by the ligand exhibiting a highly validated docking score; *S* = −5.699 Kcal·mol^−1^ with root-mean-square deviation (RMSD) relative to crystallized ligand being 1.456 Å (Figure 8A). Strong hydrogen bond pairs between the S1 sub-pocket residues, Gly143 and Ser144, and the compound’s triazole ring was predicted (Table 4). Interactions with Gly143 has been reported by irreversible and reversible inhibitors of the 3CLpro crystalline structures (PDB ID: 6Y2F, 6Y2G, 6LU7, and 6W63) [51,62]. The latter findings considered polar interactions with Gly143 significant for both covalent and non-covalent ligand-3CLpro anchoring. The predicted orientation of the ligand’s triazole ring was close enough (3.7 Å) to the Cys145 suggesting water mediated hydrogen bond interaction between such catalytic residue and ligand’s 5-membered ring. Interestingly, the trifluoromethyl substituents at C3 of the triazole scaffold suggested a conformation in vicinity towards the polar residues; His163, Glu166, and His172, lining the hydrophilic side of S1 sub-pocket. Hydrophilic interactions with the mainchain of Glu166 has been indispensable by both proteinomimetic and small molecular ligands [63,71] the ability of SIT to predict polar interaction with this key binding residues can suggest promising predicted pocket accommodation for the drug towards 3CLpro. At the other end of the docked ligand, the depicted hydrophilic interaction between the linker amide functionality and Gln189 side chain suggests further ligand-target stabilization through bounding toward the S3 sub-pocket [51,69,72,73]. Non-polar interaction was shown to be significant for anchoring the docked ligand within the 3CLpro pocket. Additionally, relevant hydrophobic contact between the ligand’s terminal phenyl moiety was predicted with S1’ sub-pocket catalytic residue, His41. The latter binding was directed through van der Walls parallel π-π stacking, suggesting the significant capability of SIT to block the catalytic activity of the enzyme.

When comparing the SIT binding mode relative to the crystallized N3 protein, pertinent comparative orientations of the ligand’s terminal phenyl and fused triazole rings was seen relative to the N3’s leucine and pyrrole residues, respectively. Moving toward the peptidomimetic ligand; MEL, significant ligand-target binding at the active site was shown to correlate to an average docking score (S = −8.618 Kcal·mol^−1^; RMSD = 3.139 Å) being almost two-folds that of SIT. Owing to extended structure and bigger size, this peptidomimetic ligand, with its 26-amino acids, depicted relevant contacts target pocket and nearby surface clefts (Figure 8B) [62,63]. However, only residues of the S3 and S1 sub-pockets offered great advantage for anchoring the MEL at the target pocket via several hydrogen bond. The key S1 sub-pocket residue, Glu166, which is responsible for anchoring many reported ligands, showed strong polar contacts with the N-terminal MEL-Lys4 side chain (1.9 Å and 125.8° as bond length and angle, respectively). Interestingly, another crucial S1 sub-pocket residue, Asp142, exhibited double hydrophilic interactions with N-terminal MEL-Gln2 side chain and C-terminal MEL-Thr10 (NHCO) main chain (Table 1). The latter confirms the significance of S1 residues in fixing both small molecules and proteinomimetic ligands at 3CLpro binding site. Anchoring of MEL at the S3 sub-pocket was mediated by a hydrogen bond pair between the side chains of both Ser46 and N-terminal MEL-Arg3. On the other hand, three polar interactions were depicted for MEL with Thr24, Glu47, and Lys61 at the target’s surface clefts beyond the S1’ sub-pocket. This, in turn, ensures the reported role of other non-sub-pocket residues for stabilizing ligand-3CLpro complex. The overlay of MEL conformation and N3 showed minimal superimposition for the Cα backbone (RMSD = 3.139 Å) owing to the larger and extended structure of the MEL protein. Nevertheless, relevant comparable orientations were depicted regarding terminal phenyl ring and pyrrole residues of N3 with several hydrophobic and polar residues of the large peptide ligand.

## 4. Discussion

In the design of nanotherapeutics, absorption is an important factor as it has a direct effect on the therapeutic load and, therefore, the effective dosage, upon entering the cells. The effectiveness of the absorption process can be influenced by changes in the physical properties of the NPs, as well as by differences in the characteristics of the cellular membrane. Pointing out the formulation and process parameters that influence the drug delivery system characteristics is fundamental in the area of pharmaceutical formulation [74,75,76,77]. Factorial design is helpful in this aspect by jointly analyzing the effect of various factors. In this study, the factors and their corresponding levels were chosen based on the results of preliminary trials. ANOVA was utilized to examine the main effects of the studied variables on each response. For both these responses, the predicted R2 values were in rational agreement with the adjusted R2 values.

Particle size could exert a significant effect on the biological performance of the nano-sized particulate delivery systems. As is evident, the size increases with an increase in both drug and MEL concentrations [78,79]. This could be attributed to increased chances for ionic interaction and aggregation of SIT-MEL complex that then leads to increased particle size. In addition, the increase in particle size is related to increased frictional forces of the entrapped SIT and MEL as their concentration increase reduces their chances of escaping and leads to increased particle size. The size of NPs is the main factor for cellular uptake and entry. The size of NPs also affects cellular internalization mechanism and intracellular localization. Peptides can be utilized to improve the uptake of NPs by cells. NPs zeta potential, a surface charge indicator, has a great impact in crossing the cell membrane (negatively charged). Additionally, NPs zeta potential has a role in improving NPs solution stability and preventing aggregation of the NPs [64,77,80]. Zeta potential indicates the charge stabilization of nanoparticulate systems. The net positive charge of SIT-Mel complex facilitates its interaction with the negatively charged phospholipids of cell membrane that, in turn, improves cellular internalization. Interestingly, we were able to prove that SIT and MEL have an antiviral activity against an isolate of SARS-CoV-2, which is synergized upon applying a mixture of the two components. 

**Figure 9:**
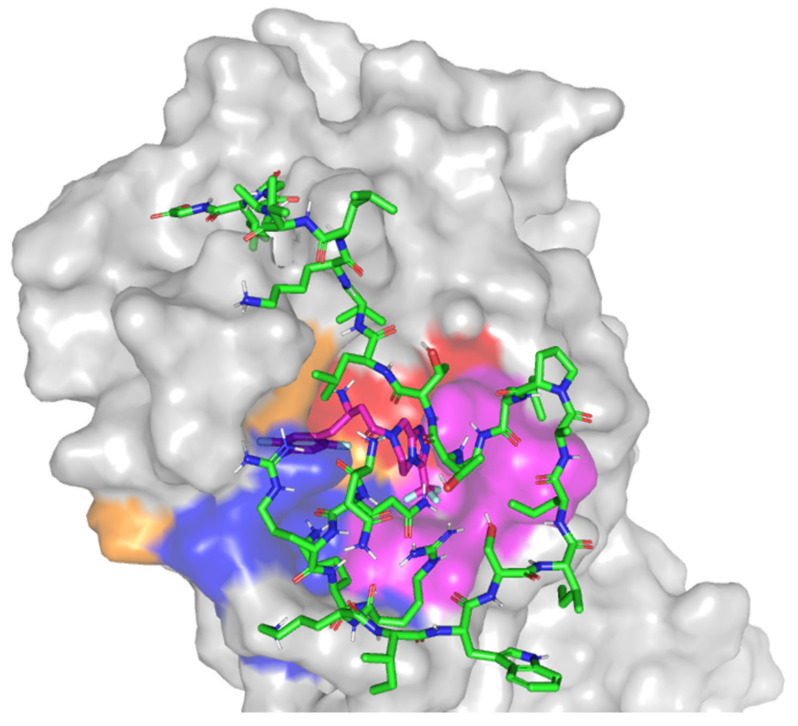
Superimposed conformations of docking ligands within SARS-CoV-2 3CL^pro^ main protease active site (PDB code: 6lu7). Docked ligands are shown as sticks 3D-representation (Melittin = green and sitagliptin = magenta) at the binding site of target protein (gray surface) representing different colored sub-pockets (S1’ = red; S1 = magenta; S2 = orange; S3 = blue).

MEL has been reported to show activity against Herpes simplex virus (HSV-1 M and HSV-2 G). MEL has shown in vitro infectivity inhibition at low concentrations (3 μM) [81]. The possible anti-viral effects of MEL on bovine herpesvirus type 1 (BoHV-1, Los Angeles strain) revealed promising results [81]. MEL (2 μg/mL) administration on Madin–Darby bovine kidney cells before and after BoHV-1 infection (MOI = 0.1) resulted in a marked reduction of viral titers. Total removal of BoHV-1 after 2-h incubation of the virus with 25 μg/mL of MEL at 37 °C, suggesting rapid anti-viral effects of MEL, was achieved with regard to virucidal kinetics [81]. In addition, the enzyme assay test using in vitro Mpro 3CL-protease was performed during our exploration for the predicted mechanism by which the complex exerts its antiviral activity. The findings revealed a very marked inhibition of Mpro 3CL-protease by the complex SIT-MEL ((IC50 = 0.3715 ± 0.001, Figure 6C), *p* < 0.001, particularly when compared to the individual components SIT (IC50 = 6.914 ± 0.034, Figure 6A) and MEL (IC50 = 1.624 ± 0.014, Figure 6B). Molecular docking study was carried out to investigate this inhibitory activity against Covid-3CL-protease enzyme. Despite their important 3CLpro active pocket accommodation, the comparative binding modes of SIT and MEL displayed very distinct orientations and binding interfaces. Compared to the much larger peptidomimetic MEL, the small-sized ligand, SIT, expected a more buried fitting inside the active site. (Figure 9). Solvent exposed residues of both S1 and S3 sub-pockets were depicted to enclose the hypoglycemic ligand away from the solvent side. Nevertheless, the obtained binding pose of MEL is more confined to the target’s pocket-solvent interface, which is highly reasoned for the ligand’s much extended structure. Importantly, stabilization of the large proteinmimietic ligand at the 3CLpro is highly mediated by extensive polar networks with relevant pocket residues, particularly Glu166 and Asn142, which have been reported by several authors as being crucial for the purpose of ligand binding. Therefore, simultaneous binding of SIT and its formulation protein, MEL, to SARS-CoV-2 3CLpro can be suggested without mutual steric hinderance impacting their respective binding mode stability [62,63,68,82].

## 5. Conclusions

The SIT-MEL complex has shown anti-viral potential against isolate of SARS-CoV-2. Moreover, according to Molecular docking, both SIT and MEL protein are capable of concurrently binding to the SARS-CoV-2 Mpro pocket within a predicted binding mode (highly stabilized) correlated to their in vitro inhibition to 3CL-protease. An optimized formulation of SIT-MEL could guarantee both enhanced delivery to the target cells and improved cellular uptake. It is recommended that biological evaluation studies are undertaken to estimate the efficiency of the optimized formulation against coronavirus 2 (SARS-CoV-2).

## Figures and Tables

**Table 1:** Independent variables and responses of SIT-MEL nano-sized systems used in 2^3^ full factorial design.

**Independent Variables**	**Levels in Coded Units**	
	**(−1)**	**(+1)**
X_1_: SIT concentration (mM)	1	10
X_2_: MEL concentration (mM)	1	10
X_3_: pH	6	10
**Responses**	**Desirability constraints**	
Y_1_: particle size (nm)	Minimize	
Y_2_: zeta potential (mV)	Maximize	

Abbreviations: SIT; Sitagliptin, MEL; Melittin, (−1); factor lower level, (+1); factor higher level.

**Table 2:** Experimental runs and the observed of responses of SIT-MEL nano-sized systems prepared in accordance with 2^3^ factorial design.

Experimental Run	Independent Variables			PS ± SD	ZP ± SD
	SIT Concentration (mM)	MEL Concentration (mM)	pH		
F-1	10	10	6	432.11± 5.12	9.11 ± 0.32
F-2	10	1	10	231.43 ± 3.21	31.21 ± 1.67
F-3	10	10	10	387.19 ± 4.91	28.35 ± 1.44
F-4	10	1	6	213.25 ± 2.98	6.27 ± 0.33
F-5	1	1	6	121.31 ± 2.11	7.19 ± 0.25
F-6	1	10	10	323.16 ± 4.99	32.25 ± 1.15
F-7	1	10	6	345.29 ± 4.31	18.39 ± 0.77
F-8	1	1	10	123.41 ± 2.11	26.42 ± 0.98

Abbreviations: SIT; Sitagliptin, MEL; Melittin, PS; particle size, ZP; zeta potential.

**Table 3:** Statistical analysis output of responses data of 2^3^ factorial design used for formulation of SIT-MEL nano-sized systems.

Responses	Process Order	*p*-Value	R^2^	Adjusted R^2^	Predicted R^2^	Adequate Precision	Significant Factors and Interactions
Y_1_: particle size (nm)	Main effects	0.0004	0.9851	0.9738	0.9405	22.21	X_1_, X_2_
Y_2_: zeta potential (mV)	2FI	0.0152	0.9999	0.9995	0.9958	116.49	X_1,_ X_2_, X_3_, X_1_X_2_, X_1_X_3_, X_2_X_3_

Abbreviations: SIT; Sitagliptin, MEL; Melittin, 2FI; two-factor interaction.

**Table 4:** Ligand-3CL^pro^ binding interaction data.

Ligand	S ^a^ (Kcal·moL^-1^)	Rescoring (Kcal·moL^-1^)	RMSD ^b^ (Å)	Ligand-Target Interaction Descriptive Data (Type; Length Å; Angle °; Binding Residues; Ligand’s Partner)
**Sitagliptin**	−5.699	−5.819	1.456	Hydrogen bond; 2.39 Å; 139.1°; Gly143 (NHCO) main chain with N1 triazole ring. Hydrogen bond; 2.37 Å; 124.6°; Ser144 (OH) side chain with N2 triazole ring Hydrogen bond; 2.31 Å; 158.1°; Gln189 (CONHH) side chain amide linker (-C=O)π-π stacking; 3.59 Å; His41 with trifluorophenyl ring
**Melittin**	−8.618	−8.821	3.139	Hydrogen bond; 2.3 Å; 121.8°; Thr24 (OH) side chain with C-terminal Melittin-Val8 (NHCO) main chain Hydrogen bond; 2.4 Å; 113.0°; Ser46 (NHCO) main chain with N-terminal Melittin-Arg3 (=NHH) side chain Hydrogen bond; 1.8 Å; 151.5°; Glu47 (COO) sidechain with C-terminal Melittin-Lys7 (NHH) side chain. Hydrogen bond; 2.2 Å; 127.8°; Lys61 (NH3+) side chain with C-terminal Melittin-Gly1 (COO) main chain Hydrogen bond; 2.2 Å; 177.2°; Asn142 (NHCO) side chain with N-terminal Melittin-Gln2 (NHH) side chain Hydrogen bond; 2.9 Å; 102.7°; Asn142 (NHCO) side chain with C-terminal Melittin-Thr10 (NHCO) main chain Hydrogen bond; 1.9 Å; 125.8°; Glu166 (NHCO) main chain with N-terminal Melittin-Lys4 (NHH) side chain

^a^ MOE docking scores using the scoring function assigned for best-ranking poses selected on the basis of visual examination and after refinement through GBVI-WSA/dG rescoring function. ^b^ Root-mean-square deviation of best-ranking pose relative to the crystallized ligand, N3.

## Data Availability

Not applicable.

## Supplementary Materials

The following are available online at https://www.mdpi.com/1999-4923/13/3/307/s1,: Table S1. design details, Table S2.design build Information, Table S3. design Factors, Table S4. design responses, Table S5. size Analysis data (ANOVA), Table S6., ZP analysis (ANOVA), Figure S1. The 3D representation of the defined SARS-CoV-2 3CLpro pocket applied within the molecular docking, Supplementary material S1: CC_50_ and IC_50_ calculation using Crystal violet assay in details., Figure S2: Illustration of plate well distribution., Supplementary Material S2: Determination of in vitro cytotoxicity, Figure S3. % cell viability of the investigated SIT (A), MEL (B) and combination of SIT-MEL (C) at different concentrations in Vero-E6 cells and expressed as % cell viability against log_10_ concentrations.
