# Supplementary material for: Repurposing of Sitagliptin- Melittin Optimized Nanoformula against SARS-CoV-2; Antiviral Screening and Molecular Docking Studies
Source: Pharmaceutics. 2021 Feb 26;13(3):307. doi: 10.3390/pharmaceutics13030307 (PMC8025909; doi:10.3390/pharmaceutics13030307)
Supplement: Supplementary file 1 [file pharmaceutics-13-00307-s001.pdf]

pharmaceutics

MDPI

Supplementary Materials: Repurposing of Sitagliptin- Melittin Optimized Nanoformula against SARS-CoV-2; Antiviral Screening and Molecular Docking Studies

Mohammed W. Al-Rabia, Nabil A. Alhakamy, Osama A. A. Ahmed, Khalid Eljaaly,Ahmed L. Aloafi, Ahmed Mostafa, Hani Z. Asfour, Ahmed A. Aldarmahi, Khaled M. Darwish, Tarek S. Ibrahim and Usama A. Fahmy

Table S1. Design details.

Std	Run	Factor 1	Factor 2	Factor 3	Response 1	Response 2
		A:SIT	B:MEL	C:pH	Size	ZP
		M	M		nm	
4	1	10	10	6	432.11	9.45
6	2	10	1	10	231.43	31.21
8	3	10	10	10	387.19	28.35
2	4	10	1	6	213.25	6.27
1	5	1	1	6	121.31	7.19
7	6	1	10	10	323.16	32.25
3	7	1	10	6	345.29	18.39
5	8	1	1	10	123.41	26.42

Abbreviations: SIT :Sitagliptin, MEL: Melittin, ZP: Zeta potential.

Citation: Al-Rabia, M.W.; Alhakamy, N.A.; Ahmed, O.A.A.; Eljaaly, K.; Mostafa, A.; Asfour, H.Z.; Aldarmahi, A.A.; Darwish, K.M.; Ibrahim, T.S.; Fahmy, U.A. Repurposing of Sitagliptin- Melittin Optimized Nanoformula against SARS-CoV-2; Antiviral Screening and Molecular Docking Studies. *Pharmaceutics* **2021**, *13*, 307. https://doi.org/10.3390/pharmaceutics13030307

Publisher's Note: MDPI stays neutral with regard to jurisdictional claims in published maps and institutional affiliations.

Copyright: © 2021 by the authors. Submitted for possible open access publication under the terms and conditions of the Creative Commons Attribution (CC BY) license (http://creativecommons.org/licenses/by/4.0/).

Table S2. Design Build Information.

File Version	11.1.2.0		
**Study Type**	**Factorial**	**Subtype**	**Randomized**
Design Type	2 Level Factorial	Runs	8
Design Model	3FI	Blocks	No Blocks
Center Points	0	Build Time (ms)	1.0000

Table S3. Design Factors.

Factor	Name	Units	Type	Minimum	Maximum	Coded Low	Coded High	Mean	Standard Deviation
A	SIT	M	Numeric	1.0000	10.00	−1 ↔ 1.00	+1 ↔ 10.00	5.50	4.81
B	MEL	M	Numeric	1.0000	10.00	−1 ↔ 1.00	+1 ↔ 10.00	5.50	4.81
C	pH		Numeric	6.00	10.00	−1 ↔ 6.00	+1 ↔ 10.00	8.00	2.14

Table S4. Design responses.

Response	Name	Units	Observations	Analysis	Minimum	Maximum	Mean	Standard deviation.	Ratio	Transform	Model
R1	Size	nm	8	Factorial	121.31	432.11	272.14	117.58	3.56	None	Main Effects
R2	ZP		8	Factorial	6.27	32.25	19.94	11.04	5.14	None	2FI

Table 5. Size Analysis data (ANOVA).

Source	Sum of Squares	df	Mean Square	*F*-Value	*p*-Value	Significance
Model	95,327.23	3	31,775.74	87.70	0.0004	significant
A-SIT	15,383.46	1	15,383.46	42.46	0.0029	
B-MEL	79,670.34	1	79,670.34	219.89	0.0001	
C-pH	273.43	1	273.43	0.7547	0.4340	
Residual	1449.28	4	362.32			
Cor Total	96,776.50	7				
**Fit statistics**						
Standard deviation				19.03		
Mean				272.14		
Coefficient of variation %				6.99		
R^2				0.9850		
Adjusted R^2				0.9738		
Predicted R^2				0.9401		
Adeq Precision				22.2134		

Table 6. ZP analysis (ANOVA).

Source	Sum of Squares	df	Mean Square	*F*-Value	*p*-Value	Significance
Model	853.09	6	142.18	2533.85	0.0152	significant
A-SIT	10.06	1	10.06	179.24	0.0475	
B-MEL	37.63	1	37.63	670.58	0.0246	
C-pH	739.78	1	739.78	13,183.84	0.0055	
AB	34.90	1	34.90	622.02	0.0255	
AC	14.45	1	14.45	257.43	0.0396	
BC	16.27	1	16.27	290.02	0.0373	
Residual	0.0561	1	0.0561			
Cor Total	853.14	7				
**Fit statistics**						
Standard deviation				0.2369		
Mean				19.94		
Coefficient of variation%				1.19		
R^2				0.9999		
Adjusted R^2				0.9995		
R^2				0.9999		
Adjusted R^2				0.9995		

(A)

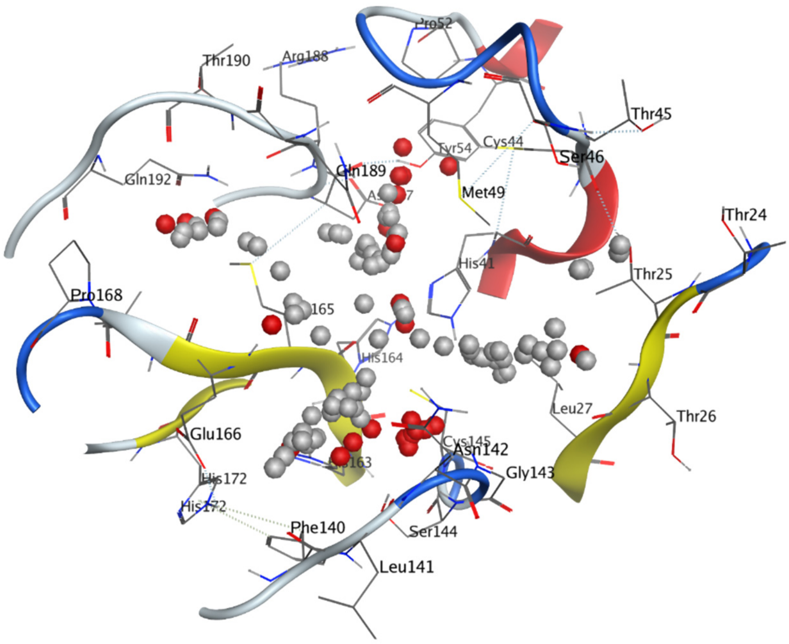

(B)

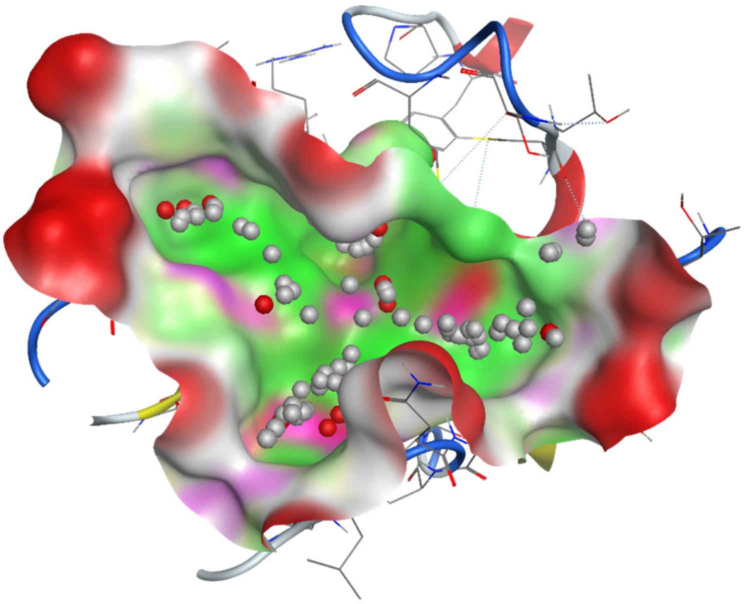

Figure S1. The 3D representation of the defined SARS-CoV-2 3CLpro pocket applied within the molecular docking study. (**A**) the docked pocket with clear representation of the contact residues (gray lines; labeled with sequence numbers), the protein's local backbone and secondary structure (gray/blue = loops; red = α-helix; yellow = β-sheets), along with the 109 alpha spheres indicates the type of atom (red = hydrophilic; white = hydrophobic) preferred by the atoms of the binding site; (**B**) molecular surface representation of the defined pocket being colored based on its hydrophobic nature of lining surfaces as well as exposure to solvent (magenta = polar; green = hydrophobic; red = solvent exposed).

Supplementary Material S1. CC_{50} and IC_{50} calculation using Crystal violet assay in details

The assay was performed according to the procedure that was previously described [1] with minor modifications. Vero E6 cells were seeded into 96-well plates in 100 µL of DMEM Complete Medium containing DMEM high glucose medium with 2 mM L-glutamine, 1 mM sodium pyruvate, and 10% fetal bovine serum (FBS), 100 units/mL penicillin and 100 µg/mL streptomycin. After 24 h (90–100% confluence monolayer of Vero E6), each compound was diluted using infection DMEM into varying concentrations in a separate U shape 96 well plate (with a range of concentration from 10 µg/mL to 1 ng/mL). An aliquot of 100 µL of each dilution was transferred into new U shape 96 well plate and supplemented with 100 $TCID_{50}$ in 100 µL infection media. In parallel the wells dedicated for CC_{50} calculation were supplemented with 100 µL infection media without virus. Aliquots of 100 µl infection media containing 100 $TCID_{50}$ were used as virus control. After 1 h of incubation, 100 µL of each well were transferred to their corresponding wells into the 96-well plates containing Vero E6 cultures (Figure X). The plates were incubated for 72 h, the cell monolayers were washed with PBS and subjected to cell fixation using 100 µL of 10% formaldehyde for 1 h. Subsequently, the plates are washed well for 3 times with 1x PBS and dried well before staining with 50 µL (0.5%) crystal violet to each well ((0.5 g crystal violet powder (Sigma-Aldrich), 80 mL distilled H_2O and 20 mL methanol)) for 30 min. the plates were then washed well with rinsed water and air-dried at room temperature for 2–24 h. To distain crystal violet, 200 µL methanol were added to each well, and the plate was incubated with its lid on a bench rocker (20 oscillations/ minute) for 20 minutes at room temperature. Finally, the optical density of each well at 590 nm (OD590) was measured with a plate reader. 100% percent was assigned to non-treated control cells, the average OD of each dilution without or with virus was compared to control cells and control virus wells to calculate % toxicity and % reduction in virus replication (respectively). The CC_{50} and IC_{50} values were calculated using nonlinear regression (three parameters) in GraphPad Prism 5.01.

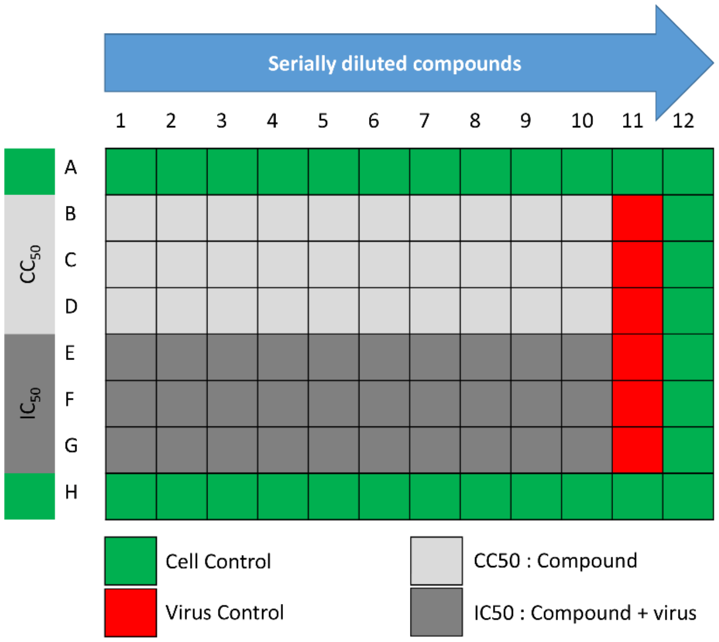

Figure 2. Illustration of plate well distribution.

Supplementary Material S2. Determination of in vitro cytotoxicity

The cytotoxic concentrations of SIT, MEL and combination of SIT-MEL in Vero-E6 cells was investigated and the result revealed that the CC50 values were 3062 and 2236 and 552.4 µg/ml, respectively (Figure S3).

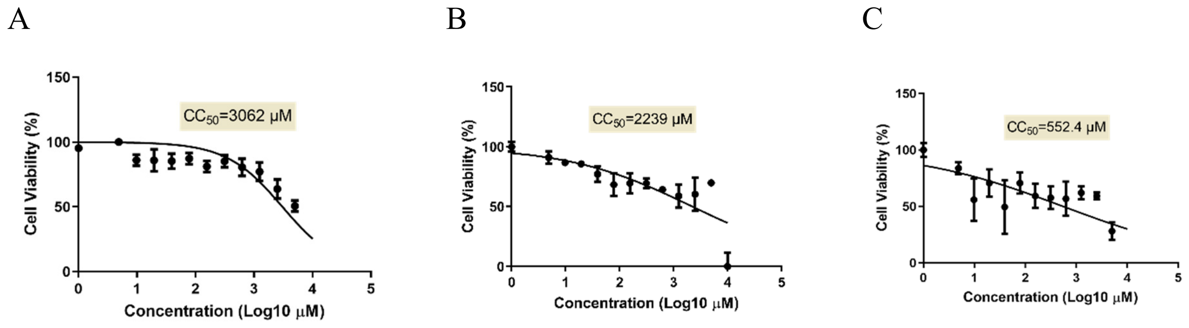

Figure S3. % cell viability of the investigated SIT (**A**), MEL (**B**) and combination of SIT-MEL (**C**) at different concentrations in Vero-E6 cells and expressed as % cell viability against log_{10} concentrations.

Reference

1. Feoktistova, M.; Geserick, P.; Leverkus, M. Crystal violet assay for determining viability of cultured cells. *Cold Spring Harb. Protoc.* **2016**, *2016*, 343–346.
